# Smart Dairy Farming: A Mobile Application for Milk Yield Classification Tasks

**DOI:** 10.3390/ani15142146

**Published:** 2025-07-21

**Authors:** Allan Hall-Solorio, Graciela Ramirez-Alonso, Alfonso Juventino Chay-Canul, Héctor A. Lee-Rangel, Einar Vargas-Bello-Pérez, David R. Lopez-Flores

**Affiliations:** 1 Computer Vision and Data Science Lab, Facultad de Ingeniería, Universidad Autónoma de Chihuahua, Circuito Universitario Campus II, Chihuahua 31125, Mexico; a358909@uach.mx; 2 División Académica de Ciencias Agropecuarias, Universidad Juárez Autónoma de Tabasco, Carr. Villahermosa-Teapa, km 25, Villahermosa 86280, Mexico; aljuch@hotmail.com; 3 Facultad de Agronomía y Veterinaria, Centro de Biociencias Universidad Autónoma de San Luis Potosí, Soledad de Graciano Sánchez 78321, Mexico; hector.lee@uaslp.mx; 4 Facultad de Zootecnia y Ecología, Universidad Autónoma de Chihuahua, Periférico R. Aldama Km 1, Chihuahua 31031, Mexico; 5 Graduate Studies and Research Division, Tecnológico Nacional de México/I.T. Chihuahua Ave. Tecnológico 2909, Chihuahua 31200, Mexico; david.lf@chihuahua.tecnm.mx

**Keywords:** milk yield classification, object detector, YOLOv11, mobile application

## Abstract

This study analyzes the use of a lightweight image-based deep learning model to classify dairy cows into low-, medium-, and high-milk-yield categories by automatically detecting the udder region of the cow. The implemented model was based on the YOLOv11 architecture, which enables efficient object detection and classification with real-time performance. The model is trained on a public dataset of cow images labeled with 305-day milk yield records. Thresholds were established to define the three yield classes, and a balanced subset of labeled images was selected for training, validation, and testing purposes. To assess the robustness and consistency of the proposed approach, the model was trained 30 times following the same experimental protocol. The system achieves precision, recall, and mean Average Precision (mAP@50) of 0.408 ± 0.044, 0.739 ± 0.095, and 0.492 ± 0.031, respectively, across all classes. The highest precision (0.445 ± 0.055), recall (0.766 ± 0.107), and mAP@50 (0.558 ± 0.036) were observed in the low-yield class. Qualitative analysis revealed that misclassifications mainly occurred near class boundaries, emphasizing the importance of consistent image acquisition conditions. The resulting model was deployed in a mobile application designed to support field-level assessment by non-specialist users. These findings demonstrate the practical feasibility of applying vision-based models to support decision-making in dairy production systems, particularly in settings where traditional data collection methods are unavailable or impractical.

## Simple Summary

This study analyzes the use of a lightweight image-based deep learning model to classify dairy cows into low-, medium-, and high-milk-yield categories by automatically detecting the udder region of the cow. Qualitative analysis revealed that misclassifications occur primarily near class boundaries, highlighting the importance of consistent image acquisition conditions. These findings demonstrate the practical feasibility of applying vision-based models to support decision-making in dairy production systems, particularly in settings where traditional data collection methods are unavailable or impractical.

## 1. Introduction

The integration of digital technologies into agriculture has transformed farming practices, resulting in the creation of more efficient, data-driven, and sustainable systems. In the context of livestock production, farmers are progressively adopting farm management systems, sensors, and electronic devices to optimize their operations, improve data collection, and enhance data-driven decision-making processes [1]. These innovations have played a significant role in the advancement of precision livestock farming, using technology to monitor and analyze various aspects of animal production. The implementation of advanced sensing systems enables farmers to obtain real-time observations of animal health, behavior, and performance. This approach has been shown to enhance productivity, improve animal welfare, and reduce labor requirements, as evidenced by recent studies [2,3,4,5].

Machine learning (ML) techniques have become essential to process the vast amounts of data generated using sensing technologies in livestock production. The review presented by Palma et al. [1] indicates that ML models—particularly artificial neural networks (ANNs) and convolutional neural networks (CNNs)—are among the most commonly used approaches for data analysis in this domain. These are followed by other methods such as random forests, support vector machines (SVMs), decision trees, fuzzy logic, k-nearest neighbors (k-NNs), and k-means clustering. Additionally, traditional statistical techniques, such as linear regression, linear discriminant analysis, and logistic regression, continue to be widely applied. Recent studies also highlight a growing trend toward the use of more advanced methods, especially deep learning architectures, to improve accuracy in tasks such as disease detection, behavioral classification, and milk yield prediction [2].

Milk yield prediction is a prominent data-driven application in the domain of livestock farming. This trait is of strategic importance, as it represents the main economic output of dairy operations [6]. Furthermore, global milk production is projected to increase steadily due to the growing demand driven by population growth and shifting dietary habits.

Dairy producers are increasingly interested in estimating the milk production of each cow early in its life, particularly when the cow is still a heifer. Precise predictions facilitate more informed decisions regarding cow replacement, culling, feeding strategies, breeding choices, financial planning, and the identification of unusual yield patterns that could signal health issues. Dairy producers need to identify the key factors that affect milk production. In doing so, they can implement effective management practices, improve milk yield, and minimize production costs.

Regarding the use of ML models to predict individual daily milk production in dairy cattle, the study by Vergani, Bagnato, and Masseroli [7] analyzes environmental variables along with phenotypic and genotypic data from 502 Holstein cows. Genetic information was obtained using the Neogen GeneSeek Genomic Profiler Bovine 100 k SNP chip. The authors compared the performance of several regression models, including AdaBoost with decision trees, ANN, k-nearest neighbors regression, linear regression, random forest regression, and linear support vector regression. Among these, the ANN model achieved the best predictive performance.

Liseune et al. [8] implemented an ANN to predict the entire lactation curve of dairy cows using historical milk yield data from the previous cycle. A Herd Management System records milkmeter information and lifetime event records such as calving, heat, pregnancy, and death in 104 different farms. The proposed architecture integrates an autoencoder, a convolutional neural network, and a multilayer perceptron. This hybrid design enables the model to capture both temporal and structural patterns in the data, thereby improving the accuracy of long-term milk yield predictions.

Angeles-Hernandez et al. [4] developed an ANN model to estimate milk yield in Pelibuey sheep based on udder measurements. The input features included initial udder circumference, final udder height, initial and final udder width, and the difference in udder volume between the initial and final measurements. The model achieved an R^2^ value of 0.93, demonstrating a high degree of predictive accuracy. Therefore, udder dimensions are useful for milk yield estimations; however, it would be more practical to do this task with a mobile application, which is not available yet.

A stacked ensemble learning model was proposed by Xing, Li, Dora, Whittaker, and Mathie [9] for predicting milk yield, utilizing biological characteristics and feeding strategies as input features. The model was trained on a dataset comprising records from 414 Holstein cattle collected between 1983 and 2019. The ensemble combined ANN, SVM, and AdaBoost as base learners, while Ridge Regression served as the meta-learner to integrate their outputs. This approach achieved an R^2^ of 0.85, highlighting the potential of meta-learning frameworks to enhance predictive performance by combining diverse machine-learning algorithms.

Gocheva-Ilieva, Yordanova, and Kulina [10] developed a model for predicting the 305-day milk yield of Holstein-Friesian cows utilizing ensemble methods, including bagging, boosting, and linear stacking predictions, to enhance the models’ predictive performance. A key aspect of the modeling process involved the development of selective ensembles, which not only reduced the number of trees within the ensemble, but also improved overall model accuracy.

The diagnosis of subclinical mastitis and the monitoring of udder health are critical for ensuring high-quality milk production. In their study, Yesil and Goncu [11] implemented an ANN that utilizes five input variables related to the physical properties of milk: somatic cell count, electrical conductivity, pH, density, and temperature measured before milking. The model was designed to classify the severity of mastitis into four categories: healthy, suspicious, subclinical, and clinical, achieving a classification accuracy of 95.1%.

Another example is reported by Bandara et al. [12], where the authors employed ML techniques and image analysis to identify cattle and classify breeds, detect parasitic diseases, and assess heat stress factors affecting milk production. Additionally, they developed a smart mobile application intended for farmers, farm managers, veterinarians, research personnel, and the general public to support cattle livestock management. The deep learning model, YOLOv3, was used to identify cattle and classify breeds. For parasite detection, fecal samples were analyzed using the YOLOv5 model. To assess heat stress levels, the authors collected data on temperature, humidity, and respiration rate, as well as categorical variables such as mouth status (open or closed) and the presence or absence of drooling saliva. These variables were manually recorded over a fixed period by a dedicated team and analyzed using a random forest algorithm.

Jembere and Chimonyo [13] implemented a deep learning model based on the Xception neural architecture to predict milk yield based on side-view and rear-view images from 743 Holstein cows. The dataset includes 1238 pairs of images, each paired with corresponding first-lactation milk yield records. The study evaluates three prediction scenarios: using only rear-view images, using only side-view images, and using a combination of both views. The resulting models achieved R^2^ values ranging from 0.30 to 0.38 and mean absolute errors between 1112.9 kg and 1148.3 kg.

As observed in the literature, various strategies have been used to predict milk yield. While advanced methods such as genomic selection can offer high predictive accuracy, their implementation across entire herds is often impractical due to high costs and limited accessibility for small- and medium-scale farms [14]. Alternatively, evaluating phenotypic traits, such as body structure and udder shape, along with environmental variables collected from different types of sensors, provides a more cost-effective option [15]. However, this approach is time-consuming and requires trained personnel with specialized expertise. Similarly, deploying multiple machine learning models to perform yield prediction can increase the complexity, computational requirements, and the need for careful model integration and validation.

To address the existing limitations, this study explores the use of 2D images as input to a deep learning model designed to classify dairy cows into low-, medium-, and high-yield milk production categories. Unlike the approach by Bandara et al. [12], which incorporates a range of sensor data beyond image-based inputs, and the method by Jembere and Chimonyo [13], which focuses on directly predicting milk yield, this work highlights the effectiveness of a purely vision-based deep learning model for the categorical classification of milk production levels. This strategy provides a cost-effective, scalable, and automated tool to support decision-making in herd management. The deep learning model employed in this study was based on the YOLOv11 architecture. YOLOv11 was selected for its ability to provide accurate object detection while maintaining low computational requirements, making it well-suited for mobile deployment scenarios. Although the model was originally designed for object detection tasks, in this study, it was trained to identify the udder region of the cow and then classify it into the appropriate yield category. This dual functionality allows for the model to focus on the most relevant anatomical region of the cow for milk production analysis. Once trained, the model is integrated into a mobile application, allowing for easy access and practical use by farmers, veterinarians, or any user interested in quickly identifying milk production potential based solely on cow images. This study represents a first step toward the automated selection of cows based on milk yield (i.e., use of a mobile application). For practical implications, this is more convenient than relying on predictions or estimations derived from traditional mathematical modeling. To our knowledge, this is one of the first studies deploying a real-time object detection model for milk yield classification using solely visual information integrated into a mobile platform.

## 2. Materials and Methods

### 2.1. Related Work

As indicated by Gunaratnam, Thayananthan, Thangathurai, and Abhiram [16], computer vision technologies have been increasingly adopted in livestock farming due to their versatility and non-invasive nature. While thermal imaging is commonly used to detect diseases in farm animals, a wide range of applications also use 2D and 3D camera sensors. These include the detection of lameness in swine and dairy cows, analysis of movement patterns in wild boars, measurement of body dimensions, detection and tracking of animals, and the evaluation of milk and meat quality, among others. Moreover, advancements in image resolution and the processing capabilities of mobile devices have facilitated the deployment of computer vision-based solutions on portable platforms, improving their practicality for on-farm use.

For instance, Aravamuthan, Walleser, and Dopfer [17] analyzed the performance of two deep learning models deployed on edge devices for the real-time detection of bovine digital dermatitis using image data. In a subsequent study, Aravamuthan, Cernek, Anklam, and Dopfer [18] conducted a comparative evaluation of six deep learning models applied to the same task. Similarly, Shorten [6] developed a computer vision system integrated with walk-over weighing technology to estimate individual cow milk yield and the lactation milk yield for individual cows. The system uses 3D images of cows and their weights measured before and after milking. A deep learning–based algorithm was used to estimate udder volume pre- and post-milking, enabling the indirect assessment of milk output.

Higaki et al. [19] proposed a pose estimation algorithm to analyze mobility in dairy cows. A total of 204 video clips of individual cows were recorded from a side-view perspective while walking. By detecting 25 anatomical key points, the authors derived 17 mobility-related variables. These features were then used as input to a random forest classifier to categorize the cows into three mobility levels: good mobility, imperfect mobility, and impaired or severely impaired mobility.

The use of ultrasonographic examination represents a non-invasive diagnostic approach widely applied in animals during on-farm evaluations. Themistokleous, Sakellariou, and Kiossis [20] presented a neural model to predict the milk yield and production stage using an ultrasound echotexture analysis of the mammary gland. Echotexture analysis was performed on the sonograms to generate a dataset containing relevant features. This set of features served as input for five ANN models designed to perform binary classification tasks, distinguishing between high lactation and no lactation, dry period vs. peak lactation, late lactation vs. peak lactation, late lactation vs. fresh cow, and peripartum vs. peak lactation, reporting an F1-score of 85.2%, 80.6%, 72.4%, 71.6%, and 65.4%, respectively.

There are limited scientific reports on the use of computer vision models for tasks related to milk yield that utilize 2D images taken with standard RGB cameras, rather than thermal imaging or ultrasound devices. Notable contributions in this area include the studies presented by Bandara et al. [12] and Jembere and Chimoyo [13]. The first study utilized images to identify cattle, classify breeds, and detect parasites in fecal samples. This visual information is combined with additional sensor data to identify factors influencing milk production. Notably, the authors developed a mobile application to make the system accessible to a wide range of users, including those without specialized expertise. In contrast, the second study performed the task as a regression problem, aiming to predict milk yield based on side- and rear-view images. However, due to the complexity of this task, the predictive performance reported in that study was limited, with R^2^ values ranging from 0.30 to 0.38 and mean absolute errors exceeding 1100 kg. Building on these insights, the present study investigates the application of a recent deep learning model to estimate milk yield as a classification problem, rather than directly predicting milk yield. Additionally, our solution was implemented as a mobile application, making it suitable for use by non-specialist users in real-world farming settings.

### 2.2. YOLOv11 Neural Architecture

**Figure 1:**
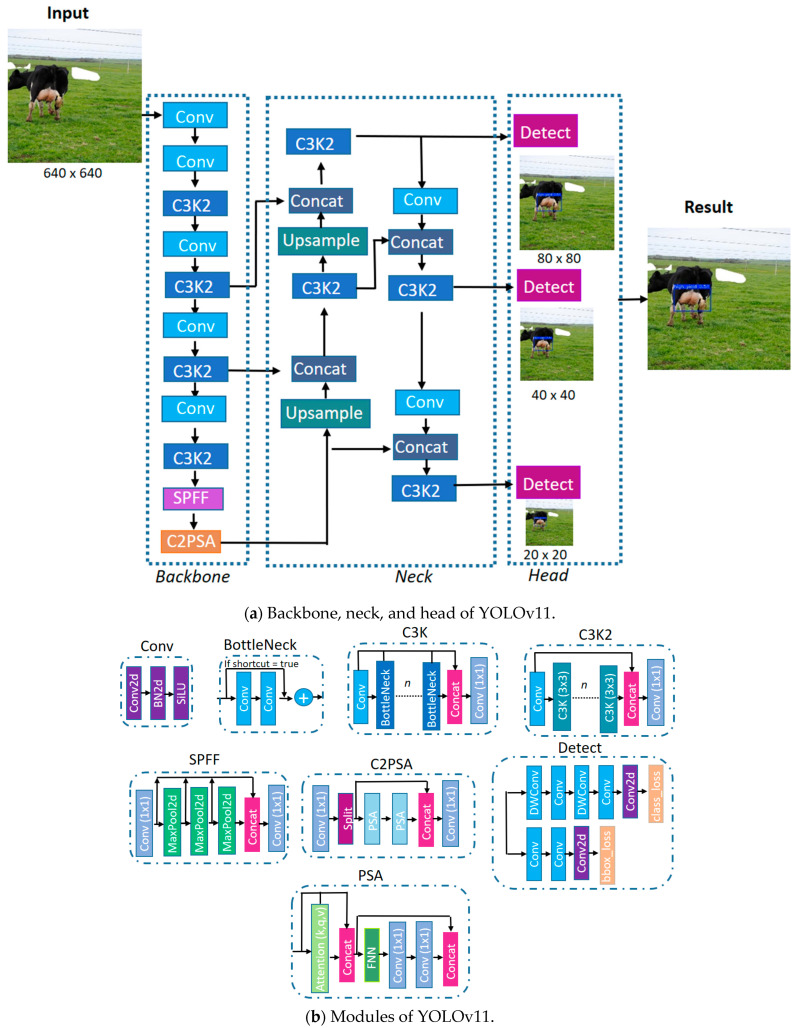
Block diagram of the YOLOv11 model. (**a**) Functional blocks representing the backbone, neck, and head responsible for extracting, combining, and interpreting features. (**b**) Detailed view of individual modules.

The YOLO family of models are well-known for their efficiency in performing object detection and classification tasks, all while featuring a lightweight architecture that is ideal for deployment on mobile devices. At the time this study was conducted, YOLOv11 [21] was the most recent version. The architecture of YOLOv11 consists of three main components, the backbone, the neck, and the head, as illustrated in Figure 1.

The backbone of YOLOv11 identifies high-level visual structures essential for accurate detection and classification. This is achieved through a series of convolutional layers that progressively reduce the spatial dimensions of the input image while increasing the number of feature channels. This process enables the extraction of rich and abstract feature representations. To enhance processing speed, the C3K2 module integrated into YOLOv11 is specifically designed for computational efficiency. It consists of several C3K sub-blocks that include BottleNeck-style structures, aimed at reducing model complexity without sacrificing performance. Within the C3K2 block, there is a configurable Boolean parameter that determines whether a residual connection is formed by concatenating the input and output of the second convolutional block. When this parameter is set to true, it improves information retention; if set to false, this connection is omitted. The last part of the backbone incorporates a Spatial Pyramid Pooling—fast (SPPF) block and the Cross Stage Partial with Spatial Attention (C2PSA) module. These components help the model focus on important spatial areas within the image, thereby enhancing the extraction of relevant features.

The neck of the model combines the feature maps extracted at different scales from the backbone. It uses upsampling operations and feature concatenation to integrate both low-level and high-level information. During this process, C3K2 blocks are interspersed to improve multiscale feature integration. The resulting fused representations are then sent to the head module, which produces the final detection outputs.

The head is the final stage of architecture and is responsible for generating the model’s predictions. It processes the multiscale feature maps received from the neck to simultaneously produce the bounding boxes and class labels for objects within the image.

A key concept in evaluating the YOLOv11 neural architecture is the Intersection over Union (IoU) metric. IoU quantifies the overlap between a predicted bounding box and its corresponding ground truth bounding box. It is calculated as the ratio of the area of overlap to the area of the union of the two boxes, as illustrated in Equation (1).

(1) $$\mathrm{IoU}=\frac{\mathrm{Area}\;\mathrm{of}\;\mathrm{Overlap}}{\mathrm{Area}\;\mathrm{of}\;\mathrm{Union}}$$

**Figure 2:**
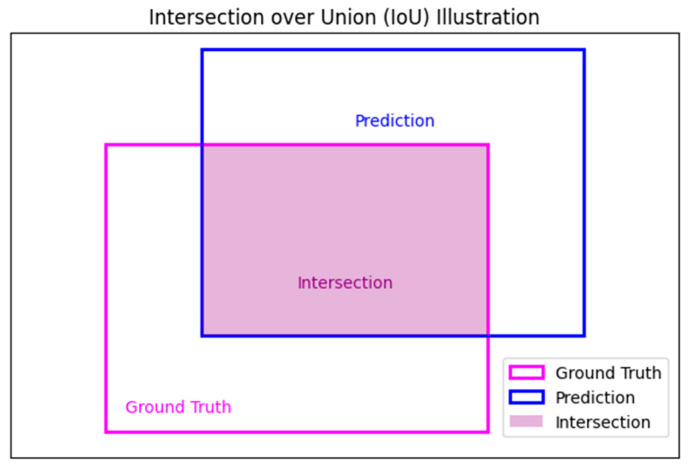
Graphical representation of IoU calculation based on the overlap between predicted and ground truth bounding boxes.

IoU values range from 0 to 1. An IoU of 1 indicates a perfect match between the predicted bounding box and the ground truth box, while an IoU of 0 signifies no overlap at all. In object detection tasks, a prediction is considered a true positive (*TP*) if its IoU with a ground truth box exceeds a predefined threshold, which is commonly set at 0.5. If the IoU does not exceed this threshold, the prediction is classified as a false positive (*FP*). Figure 2 provides a graphical representation of how the IoU was calculated.

YOLOv11 uses the mean Average Precision (mAP) as a metric to evaluate the performance of the model. mAP is calculated as follows:

(2) $$\mathrm{Precision}=\frac{TP}{TP+FP}$$

(3) $$\mathrm{Recall}=\frac{TP}{TP+FN}$$

(4) $$AP=\int_{0}^{1}\mathrm{Precision}(r)dr$$

(5) $$\mathrm{mAP}=\frac{1}{N}\sum_{i=1}^{N}AP_{i}$$

A prediction is considered a *TP* only if both the bounding box localization and the class label are correct, based on the defined Intersection over Union (IoU) threshold. Precision (2) measures the proportion of positive predictions made by the model that are correct. In other words, it reflects how many of the predicted objects belonging to a given class truly do belong to it. High precision indicates that the model rarely mislabels objects, as it minimizes the number of false positives (*FP*s). In contrast, recall (3) measures the proportion of actual objects that are correctly detected by the model. High recall is particularly important in scenarios where missing an object can have serious consequences, such as detecting a disease, where it is preferable to capture all potential cases, even at the cost of some false negatives. Here, false negatives (*FN*s) refer to ground truth objects that the model fails to detect. Both precision and recall are used to compute the Average Precision (*AP*) and the mean Average Precision (mAP). *AP* corresponds to the area under the precision–recall curve, with higher values indicating more consistent and reliable detection for a given class. *r* denotes the recall value, and precision(*r*) represents the corresponding precision. The mAP is calculated as the mean of the *AP* values across all object classes, where *N* is the total number of classes and *APᵢ* is the Average Precision for class *i*.

YOLOv11 was selected over models such as Faster R-CNN [22], EfficientDet [23], and earlier YOLO versions due to its proven suitability for mobile deployment in real-world conditions [24,25]. While some of these alternatives may offer higher accuracy, they typically require greater computational resources. In many cases, they also rely on compression techniques that can degrade performance. In contrast, YOLOv11 offers a balanced trade-off between accuracy and efficiency. This is achieved through architectural improvements such as C3K2 blocks, the SPPF module, and the C2PSA attention mechanism, which enhance spatial focus and computational performance. These features make it particularly well-suited for edge-level applications.

## 3. Results

The dataset used in this study was obtained from Kaggle and is publicly available at https://www.kaggle.com/datasets/lawrencejembere/cow-images-for-milk-yield-prediction (accessed on 20 March 2025). This dataset includes rear-view and side-view images of the cows, along with their corresponding 305-day milk yield records from the first lactation. All cows were born in 2017, 2018, or the early months of 2019. They were managed within a pasture-based production system and were milked twice daily. Morning milking started at either 03:00 or 04:00 h, while afternoon milking took place at either 14:00 or 15:00 h, depending on the specific farm. The images were obtained from cows in their first or second lactation. Image acquisition was carried out between 07:00 and 18:00 h, capturing cows on various days in milking and at different times relative to milking, irrespective of lactation stage. This strategy introduced variability in udder fullness, which fluctuates significantly throughout the day based on milking schedules. Image acquisition was performed when cows were grazing in pasture environments, a context that reduced potential disruptions from routine farm operations and promoted a calmer behavioral state among the animals, thereby optimizing conditions for consistent and reliable image capture. The distance between the photographer and the cow varied across images to introduce scale variability.

**Figure 3:**
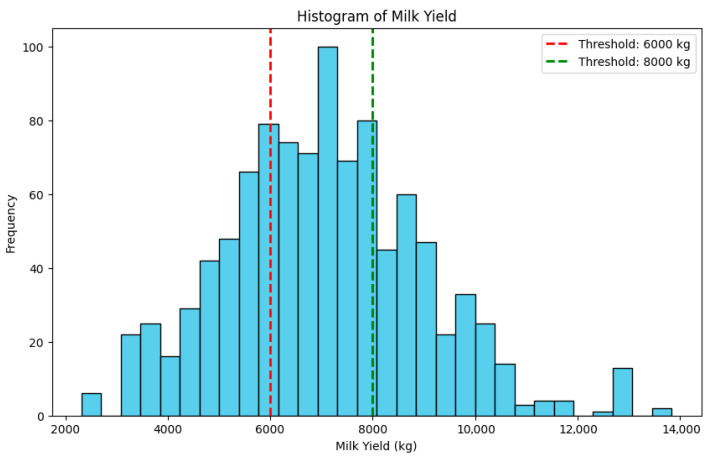
Milk yield distribution with thresholds for low-, medium-, and high-milk yields.

For the classification task addressed in this study, the milk yield distribution of the dataset was analyzed to establish threshold values for categorizing cows into low-, medium-, and high-production classes. Specifically, cows producing less than 6000 kg of milk are labeled as low producers, those producing between 6000 kg and 8000 kg as medium producers, and those exceeding 8000 kg as high producers. The thresholds are determined using a histogram analysis of the milk yield data. As shown in Figure 3, the distribution is right-skewed, with most values ranging between 6000 kg and 8000 kg. The selected cutoffs correspond to the lower and upper quartiles, ensuring both class balance and meaningful separation between yield categories.

Based on these criteria and to maintain a balanced dataset, 300 cows are randomly selected for the low-production class, 298 for the medium-production class, and 295 for the high-production class. Table 1 presents the data distribution used for the training, validation, and testing of the YOLOv11 model.

The image annotation process for the classification and detection system is conducted using the Roboflow platform. All bounding boxes corresponding to the udder region are initially labeled by a single annotator trained in livestock image interpretation. To ensure annotation consistency and reduce subjectivity, two additional reviewers independently verified the annotations. Any discrepancies were resolved through consensus. Once fully annotated, the dataset was used to train the YOLOv11 model to perform both detection of the udder anatomical region and classification into the appropriate milk yield category. These tasks were implemented using the Ultralytics library. The training was configured for a total of 400 epochs; however, the model reached satisfactory performance and converged early at epoch 187, thus reducing unnecessary computational effort. A batch size of 8 was used, and all input images were resized to a resolution of 640 × 640 pixels. The model was initialized with pre-trained weights from the YOLO11n.pt checkpoint, which accelerates convergence and improves training stability during the early stages. The optimization process employed the AdamW optimizer with a learning rate of 0.01 and a momentum value of 0.937. To mitigate overfitting, early stopping based on validation loss was implemented during training. This method automatically halts the training process when performance on the validation set stops improving, thereby reducing the risk of the model overfitting to the training data. Additionally, batch normalization layers inherent to the YOLOv11 architecture and data shuffling during training contributed to more stable convergence and improved generalization. To evaluate the consistency and robustness of the proposed model, the training process was repeated 30 times using the same dataset partitioning and configuration [26]. Each run was initialized with a different random seed to introduce variability in weight initialization and data shuffling. This approach enables a statistical analysis of model performance across runs and provides insight into the stability and generalization capacity of the system. All experiments are conducted on a Linux-based operating system using an NVIDIA RTX 4080 GPU with 16 GB of dedicated VRAM and 64 GB of system memory.

**Figure 4:**
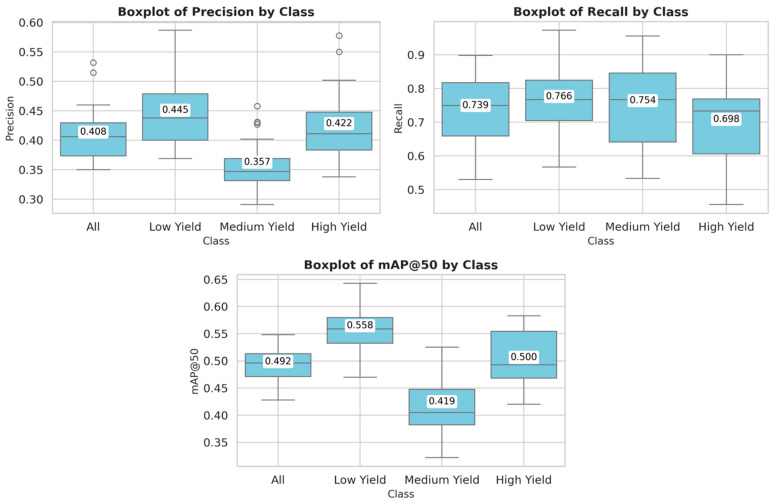
Distribution of precision, recall, mAP@50, and mAP@50–95 across yield categories (all, low, medium, and high yield) over 30 independent training runs.

Table 2 shows the evaluation metrics obtained on the test set across 30 independent training runs, reporting the mean and standard deviation for each milk yield class. The metric mean Average Precision at an Intersection over Union threshold of 0.50 (mAP@50) reflects the average detection performance when bounding boxes exceed this threshold. These trends are further illustrated in Figure 4, which shows box plots of the aforementioned metrics for each milk yield class. The visualizations confirm the superior performance of the model in the low-yield category.

Among all categories, the low-yield class consistently achieves the highest performance, with a precision of 0.445 ± 0.055, a recall of 0.766 ± 0.107, and mAP@50 of 0.558 ± 0.036, indicating that the model is most accurate and reliable in identifying cows within this group. In contrast, the medium-yield class shows the lowest values for precision (0.357 ± 0.042) and mAP@50 (0.419 ± 0.049), despite a relatively high recall (0.754 ± 0.129), suggesting a tendency toward over-detection and a higher rate of false positives. The high yield class presents intermediate performance, with a balanced trade-off between precision and recall. Overall, the average metrics across all classes are 0.408 ± 0.044 for precision, 0.739 ± 0.095 for recall, and 0.492 ± 0.031 for mAP@50.

**Figure 5:**
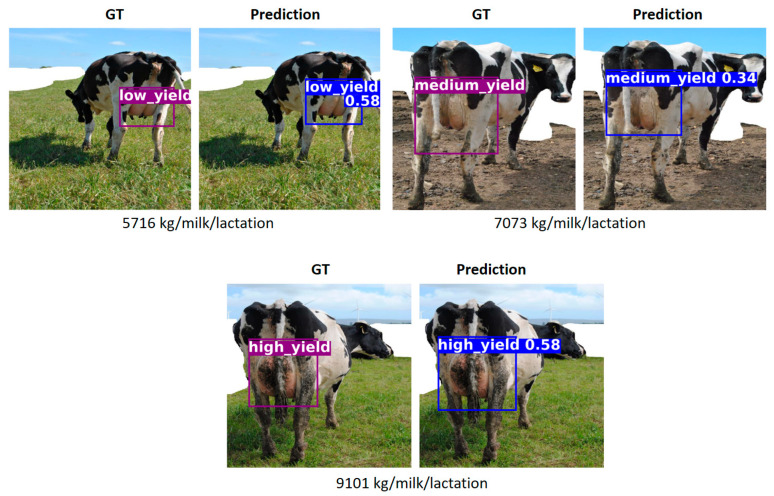
Examples of correct predictions made by the implemented model.

Figure 5 shows qualitative results from a single representative run, illustrating the implemented model’s predictions for different yield classes. Each row includes the ground truth (GT) and the corresponding prediction for a given cow. The actual milk yield is shown below each pair of images. In all three examples, the model correctly classifies the cow into its respective production category.

**Figure 6:**
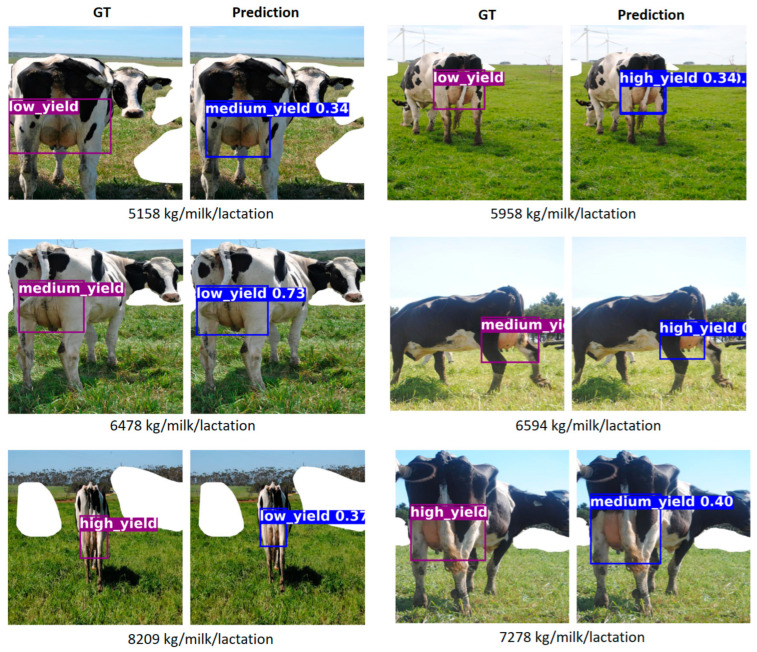
Examples of erroneous predictions.

Figure 6 shows examples of misclassifications from a single representative run of the implemented model, illustrating the typical errors observed across yield categories. The image pairs are organized to highlight common patterns of confusion between classes. In the first row, the ground truth corresponds to the low-yield class; however, the model misclassifies these cows as medium- (left pair) and high-yield (right pair). It is worth noting that the actual milk yields of these cows are near the upper threshold of the low-yield category, which may have contributed to the misclassification, particularly in the first pair, where the predicted class closely borders the ground truth. The next row shows examples of cows labeled as medium-yield. In these cases, the model incorrectly assigns the low-yield class (left pair) and the high-yield class (right pair), respectively. These samples also lie near the lower threshold of the medium yield category, which may introduce visual ambiguity, particularly in the first pair, where the cow’s appearance could plausibly resemble that of a low-yield animal. In the second case, visual inspection suggests that the cow might reasonably be perceived as a high-yield producer, which may have influenced the model’s prediction. The last row depicts high-yield cows that were incorrectly classified as low-yield (left pair) and medium-yield (right pair). Again, visual inspection reveals that these cows exhibit features that could lead to confusion. Overall, these examples illustrate that most misclassifications occur in cases where the actual milk yield is close to the defined thresholds between classes or where the visual appearance of the cow may reasonably resemble that of the predicted class, even if it does not match the ground truth label.

To provide real-time predictions based on images captured directly using a smartphone camera, a mobile application was developed using Ultralytics HUB. This cloud-based platform supports the training, management, and deployment of YOLO models on edge devices such as mobile phones. In this study, Ultralytics HUB is explicitly used for deployment, allowing for the integration of the pre-trained YOLOv11 model into a mobile environment. The platform supports both Android and iOS and enables offline inference, making it suitable for use in field conditions.

**Figure 7:**
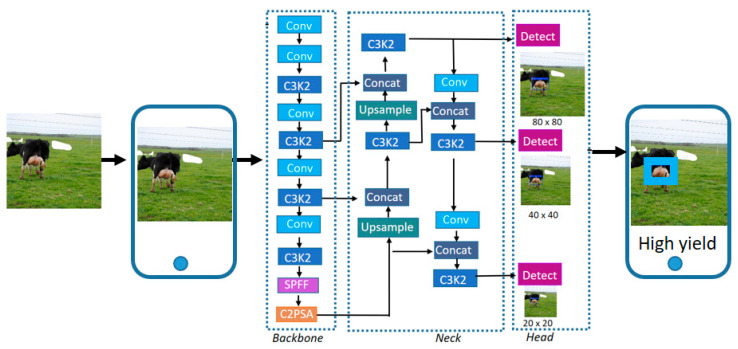
Block diagram of the implemented mobile-based milk yield classification approach.

The trained model achieves an average processing speed of 21.8 frames per second (fps) on an Android device, corresponding to an inference time of approximately 46.4 milliseconds per frame. These results demonstrate that the system is capable of real-time operation, providing fast and responsive classification. Figure 7 presents a block diagram of the mobile-based deployment pipeline, illustrating the main components involved in image acquisition, model inference, and result display within the application.

## 4. Discussion

This study assesses the effectiveness of the YOLOv11 deep learning model to classify dairy cows into low-, medium-, and high-yield milk production groups by detecting the udder region of the cow. To our knowledge, this study represents one of the first real-time applications of object detection for milk yield classification based solely on visual features, deployed via a mobile interface for practical use in the field. As a recent variant of the YOLO architecture, YOLOv11 incorporates several enhancements—such as C3K2, SPPF, and C2PSA—that improve spatial attention and computational efficiency, making it well-suited for real-time deployment on mobile devices.

The model shows particularly strong performance in the low-yield category, achieving the highest mean precision, recall, and mAP@50 across 30 independent training runs. These results suggest a high degree of reliability in identifying cows in this group, likely due to more distinctive visual characteristics associated with lower milk production. In contrast, the medium-yield category proves to be the most challenging, with the lowest precision and mAP scores, despite relatively high recall. This indicates that, while the model successfully detects many medium-yield cows, it frequently misclassifies samples from other categories as medium yield, leading to a higher rate of false positives.

From a practical perspective, these patterns remain useful: accurate identification of low-yield cows enables timely decisions related to culling or management interventions, while the high recall in the medium-yield group may still be beneficial for early-stage screening scenarios where broader detection is acceptable.

Qualitative results offer further insight into the observed misclassifications. A significant number of errors occur when the actual milk yield falls near the boundaries defined by the quartile-based thresholds. Additionally, the analysis reveals that some misclassifications stem from visual ambiguity cases where a cow’s appearance closely resembles that of a low-yield animal, despite belonging to a different category. This suggests that certain visual features are shared across classes, particularly near the threshold regions, which complicates accurate classification.

Overall, the results confirm the practical feasibility of using lightweight deep learning models to support decision-making in herd management, particularly in resource-constrained or field-based environments. However, the effectiveness of such models is highly dependent on the quality and representativeness of the annotated dataset. Improving classification performance, particularly for the intermediate (medium yield) category may require the inclusion of more diverse and balanced training samples, along with enhanced annotation strategies that more accurately capture the subtle visual differences between classes.

Compared to previous studies focused on milk yield prediction using machine learning techniques [4,7,8,9,10,11], our approach eliminates the need for manual measurements, specialized equipment, or expert intervention. Although the implementation of machine learning models is generally straightforward, obtaining the appropriate input data often poses a significant challenge. In this context, our image-based classification strategy streamlines data acquisition by relying solely on images of the cows, offering a non-invasive and more accessible alternative.

Moreover, in line with findings reported in [6,16,17,18,19,20], the integration of computer vision and deep learning in the agricultural sector has demonstrated considerable potential, particularly when implemented on edge devices, as shown in [12,17]. Our study contributes to this growing field of research by demonstrating the feasibility of deploying a lightweight milk yield classification system on mobile platforms, thereby improving accessibility, scalability, and practicality for use in real farm conditions.

Nonetheless, future work should consider refining the classification thresholds through consultation with domain experts to reflect real-world production boundaries more accurately. Additionally, it is important to establish a robust methodology for image acquisition, particularly in mobile application scenarios. Some incorrect predictions observed in this study were likely caused by variations in image capture distance; images taken either too close or too far from the animal affected model performance. Notably, several of these misclassified cases may also be visually ambiguous to human observers, especially when the cow’s appearance is not strongly indicative of its actual milk yield. Therefore, clear guidelines for proper image capture should be defined to ensure the consistent and reliable use of the tool in practical settings. This study also demonstrates the viability of deploying a lightweight milk yield classification system on mobile devices.

As a continuation of this research, we propose collaborating with local dairy producers in Latin America who have expressed interest in adopting this technology. The goal is to build a region-specific dataset validated by multiple domain experts, which will not only strengthen model training, but also enhance its applicability and trustworthiness in real-world farm conditions. Additionally, to overcome the domain limitations of the current dataset, which is restricted to a specific breed and production system, future work will focus on fine-tuning the model using previously labeled images from the target cattle populations. This retraining phase will enable the model to adapt to the distinct visual characteristics of different breeds and production environments, thereby enhancing its generalizability and supporting reliable deployment across a wider range of farm settings.

## 5. Conclusions

This study evaluates the effectiveness of the YOLOv11 deep learning model in classifying dairy cows into low-, medium-, and high-yield milk production categories by detecting the udder region. Across 30 independent training runs, the model achieved a mean mAP@50 of 0.492 ± 0.031 for all classes. The best performance was observed in the low-yield category, with a precision of 0.445 ± 0.055 and a recall of 0.766 ± 0.107.

To demonstrate the model’s suitability for mobile deployment, one of the trained versions was integrated into a mobile application using the Ultralytics HUB platform. This enables real-time offline inference on Android devices, confirming the model’s practical utility in field conditions. The resulting app leverages advanced image recognition and deep learning to estimate milk yield from a simple photograph, eliminating the need for invasive or labor-intensive methods. By combining scientific accuracy with user-friendly technology, it empowers farmers to make fast, data-driven decisions directly in the field.

Future work will focus on enhancing class separability, expanding the dataset to include a wider variety of breeds and production systems, and evaluating the app under diverse real-world conditions to support broader adoption.

## Figures and Tables

**Table 1:** Data distribution across training, validation, and test sets based on milk yield.

	Train	Valid	Test	Total
Low	210	60	30	300
Medium	209	59	30	298
High	206	59	30	295
Total	625	178	90	893

**Table 2:** Evaluation metrics obtained on the test set for each milk yield class.

Class	Precision(P)	Recall(R)	mAP@50
All	0.408 ± 0.044	0.739 ± 0.095	0.492 ± 0.031
Low yield	0.445 ± 0.055	0.766 ± 0.107	0.558 ± 0.036
Medium yield	0.357 ± 0.042	0.754 ± 0.129	0.419 ± 0.049
High yield	0.422 ± 0.058	0.698 ± 0.123	0.500 ± 0.049

## Data Availability

The data presented in this study are available on request from the corresponding author.
